# Supplementary material for: Interplay of Crystallization and Amorphous Spinodal Decomposition During Thermal Annealing of Organic Photoactive Layers
Source: Adv Sci (Weinh). 2026 Mar 9;13(26):e24140. doi: 10.1002/advs.202524140 (PMC13159141; doi:10.1002/advs.202524140)
Supplement: Supplementary file 1 — Supporting File: advs74644‐sup‐0001‐SuppMat.pdf. [file ADVS-13-e24140-s001.pdf]

Interplay of Crystallization and Amorphous Spinodal Decomposition during Thermal Annealing of Organic Active Layers

Supplementary Information

Maxime Siber,*[a,b] Olivier J. J. Ronsin,[a] Gitti L. Frey,[c] and Jens Harting [a,b,d]

[a] *Helmholtz Institute Erlangen-Nürnberg for Renewable Energy, Forschungszentrum Jülich, Fürther Straße 248, 90429 Nürnberg, Germany, E-mail: m.siber@fz-juelich.de*
[b] *Department of Chemical and Biological Engineering, Friedrich-Alexander-Universität Erlangen-Nürnberg, Fürther Straße 248, 90429 Nürnberg, Germany*
[c] *Department of Material Science and Engineering, Technion Israel Institute of Technology, Haifa 3200003, Israel*
[d] *Department of Physics, Friedrich-Alexander-Universität Erlangen-Nürnberg, Fürther Straße 248, 90429 Nürnberg, Germany*

Contents

A Phase-Field Model - Free Energy Formulation 1

B Phase-Field Model - Transport Equations 3

C Phase-Field Model - Parameter Tables 4

D Effect of Blend Ratio on Spinodal Decomposition Morphology 8

E Effect of Surface Tension on Crystal Size and Density 9

F Effect of Diffusion-Limited Growth on Crystal Arrangement 11

G Effect of PCE11 Crystallites - Additional Simulations 16

References 18

A Phase-Field Model - Free Energy Formulation

In the context of Phase-Field modelling, the Gibbs free energy of the system that is sought to be represented is expressed as a functional G which involves several free energy density contributions (here $G_V^{(bulk,ac)}$, $G_V^{(bulk,mix)}$, $G_V^{(grad,ac)}$, $G_V^{(grad,mix)}$, $G_V^{(ori,ac)}$, and $G_V^{(num,mix)}$):

$$G = \int \left[G_V^{(bulk,ac)} + G_V^{(bulk,mix)} + G_V^{(grad,ac)} + G_V^{(grad,mix)} + G_V^{(ori,ac)} + G_V^{(num,mix)}\right] dV \ . \quad (1)$$

All these contributions are functions of so-called field variables that are local descriptors of phase properties and vary continuously throughout the system, inherently giving rise to diffuse interfaces between different phase domains. To monitor phase separation phenomena occurring within the investigated PCE11:PCBM mixture, the model relies on the volume fractions of both components (ϕ_{PCE11} and ϕ_{PCBM}) as compositional field variables. Since $\phi_{\mathrm{PCE11}} + \phi_{\mathrm{PCBM}} = 1$ (i.e. $\phi_{\mathrm{PCBM}} = 1 - \phi_{\mathrm{PCE11}}$), only one volume fraction needs to be kept track of in practice. In what follows, ϕ_{PCE11} is used.
To quantify the progress of the phase transitions undergone by the mixed materials from the amorphous to the crystalline state, the model makes use of structural order parameters (ψ_{PCE11} and ψ_{PCBM}) which range from 0, meaning that the species is fully amorphous, to 1, indicating that it is completely crystallized. Conversely to the volume fractions, the order parameters ψ_{PCE11} and ψ_{PCBM} are independent, as PCE11 crystallization and PCBM crystallization are two separate processes (although they can still interfere with each other).
The related crystallization free energy contribution arising within the homogeneous bulk of a phase ($G_V^{(bulk,ac)}$) writes

$$
\begin{aligned}
G_V^{(bulk,ac)} =& \phi_{\mathrm{PCE11}}\rho_{\mathrm{PCE11}}\left[q(\psi_{\mathrm{PCE11}})W_{\mathrm{PCE11}} + p(\psi_{\mathrm{PCE11}})L_{\mathrm{PCE11}}\left(\frac{T}{T_{m,\mathrm{PCE11}}} - 1\right)\right] \\
&+ (1-\phi_{\mathrm{PCE11}})\,\rho_{\mathrm{PCBM}}\left[q(\psi_{\mathrm{PCBM}})W_{\mathrm{PCBM}} + p(\psi_{\mathrm{PCBM}})L_{\mathrm{PCBM}}\left(\frac{T}{T_{m,\mathrm{PCBM}}} - 1\right)\right] .
\end{aligned} \tag{2}
$$

with ρ_{PCE11} and ρ_{PCBM} standing for the densities of both species, L_{PCE11} and L_{PCBM} representing their respective latent heats of fusion, $T_{m,\mathrm{PCE11}}$ and $T_{m,\mathrm{PCBM}}$ denoting their melting temperatures, and W_{PCE11} and W_{PCBM} designating the energy barrier coefficients that need to be overcome during nucleation to form stable crystals. Moreover, T is the temperature of the system. The quantities $p(\psi_{\mathrm{PCE11}})$, $p(\psi_{\mathrm{PCBM}})$, $q(\psi_{\mathrm{PCE11}})$, and $q(\psi_{\mathrm{PCBM}})$ are polynomials that interpolate the crystallization free energy based on the values of the order parameters ψ_{PCE11} and ψ_{PCBM} and ensure that $G_V^{(bulk,ac)}$ overall retains a double-well shape suited to adequately model the crystallization process. Multiple different functions are reported for this purpose with the Phase-Field approach [1, 2]. In this work, the following polynomial forms are employed,

$$
\begin{cases}
p(\psi_i) & = \psi_i^2(3-2\psi_i) \ , \\
q(\psi_i) & = \psi_i^2(1-\psi_i)^2 \ ,
\end{cases} \tag{3}
$$

where the subscript index i indiscriminately refers to any of the blended species.

As a crystal germ materializes within the system, surface tension also manifests at its interfaces. An interface between a crystal and its amorphous surroundings is characterized by a gradient in order parameter ψ_{PCE11} or ψ_{PCBM}. The associated free energy contribution $G_V^{(grad,ac)}$ reads

$$
G_V^{(grad,ac)} = \frac{\varepsilon^2_{\mathrm{PCE11}}}{2}|\nabla\psi_{\mathrm{PCE11}}|^2 + \frac{\varepsilon^2_{\mathrm{PCBM}}}{2}|\nabla\psi_{\mathrm{PCBM}}|^2 \ , \tag{4}
$$

with $\varepsilon^2_{\mathrm{PCE11}}$ and $\varepsilon^2_{\mathrm{PCBM}}$ the surface tension coefficients of PCE11 and PCBM crystals, respectively.

Additionally, modelling polycrystalline systems requires to handle the eventual impingement of distinct crystal seeds. For this, an orientation parameter θ is attributed individually to all stable crystal nuclei. A Dirac delta function $\delta(\nabla\theta)$ is used to detect the location where the orientation changes over a crystal-crystal interface (i.e. the impingement location), thereby activating the crystal impingement free energy contribution ($G_V^{(ori,ac)}$) defined as

$$
G_V^{(ori,ac)} = p(\psi_{\mathrm{PCE11}})\frac{\alpha_{\mathrm{PCE11}}}{2}\delta(\nabla\theta) + p(\psi_{\mathrm{PCBM}})\frac{\alpha_{\mathrm{PCBM}}}{2}\delta(\nabla\theta) \ . \tag{5}
$$

The coefficients α_{PCE11} and α_{PCBM} then regulate the strength of this contribution weighted by the extent of the crystallization phase transition at the impingement location (respectively evaluated with $p(\psi_{\mathrm{PCE11}})$ and $p(\psi_{\mathrm{PCBM}})$).

Along with the physics of crystallization, the free energy functional captures material interactions arising upon mixing of PCE11 and PCBM. The expression of the related mixing free energy contribution from the bulk ($G_V^{(bulk,mix)}$) stems from the Flory-Huggins theory for amorphous polymer mixtures [3–5] and its extension by Matkar and Kyu to account as well for crystalline components [6, 7]:

$$
\begin{aligned}
G_V^{(bulk,mix)} =& \frac{RT}{v_0}\left[\frac{\phi_{\mathrm{PCE11}}}{N_{\mathrm{PCE11}}}\ln(\phi_{\mathrm{PCE11}}) + \frac{(1-\phi_{\mathrm{PCE11}})}{N_{\mathrm{PCBM}}}\ln(1-\phi_{\mathrm{PCE11}}) \right. \\
&\left. + \phi_{\mathrm{PCE11}}(1-\phi_{\mathrm{PCE11}})\left(\chi^{(aa)} + \chi^{(ca)}\psi^2_{\mathrm{PCE11}} + \chi^{(ac)}\psi^2_{\mathrm{PCBM}} + \chi^{(cc)}\psi_{\mathrm{PCE11}}\psi_{\mathrm{PCBM}}\right)\right] .
\end{aligned} \tag{6}
$$

Here, R is the ideal gas constant, v_0 represents the molar volume of the Flory-Huggins lattice sites, and N_{PCE11} and N_{PCBM} indicate the sizes of both blend components in terms of these lattice elements (so that the products $v_0 N_{\mathrm{PCE11}}$ and $v_0 N_{\mathrm{PCBM}}$ yield the molar volumes of PCE11 and PCBM). $\chi^{(aa)}$ is the classical Flory-Huggins interaction parameter which characterizes the miscibility of the species in the amorphous state and is empirically expected to take the following form [5]:

$$
\chi^{(aa)} = A + \frac{B}{T} \ , \tag{7}
$$

where A is an entropy and B/T an enthalpy contribution. According to Matkar and Kyu [6, 7], the parameters resulting from interactions of crystalline PCE11 with amorphous PCBM (χ_{ca}), and crystalline PCBM with amorphous PCE11 (χ_{ac}), are proportional to the crystallization enthalpies L_{PCE11} and L_{PCBM}, respectively, that is

$$\chi^{(ca)} = C_{\rm PCE11}\frac{v_0 N_{\rm PCE11}\rho_{\rm PCE11}L_{\rm PCE11}}{RT}\text{ , and }\chi^{(ac)} = C_{\rm PCBM}\frac{v_0 N_{\rm PCBM}\rho_{\rm PCBM}L_{\rm PCBM}}{RT}\ . \tag{8}$$

The constants $C_{\rm PCE11}$ and $C_{\rm PCBM}$ denote the corresponding proportionality coefficients.
Surface tension also acts at interfaces between phases of different compositions. A free energy contribution ($G_V^{(grad,mix)}$) similar to $G_V^{(grad,ac)}$ scales quadratically with the magnitude of the gradient in volume fraction $\nabla\phi_{\rm PCE11}$, and linearly with $\kappa_{\rm PCE11}$ and $\kappa_{\rm PCBM}$, the surface tension coefficients pertaining to both blend components:

$$G_V^{(grad,mix)} = \frac{\kappa_{\rm PCE11}}{2}|\nabla\phi_{\rm PCE11}|^2 + \frac{\kappa_{\rm PCBM}}{2}|\nabla(1-\phi_{\rm PCE11})|^2 = \frac{\kappa_{\rm PCE11}+\kappa_{\rm PCBM}}{2}|\nabla\phi_{\rm PCE11}|^2\ , \tag{9}$$

Finally, the free energy model also includes a numeric regularization term $G_V^{(num,mix)}$ that prevents solver instabilities when the volume fraction $\phi_{\rm PCE11}$ reaches values close to 0 and 1:

$$G_V^{(num,mix)} = \frac{\beta}{\phi_{\rm PCE11}^{\gamma}} + \frac{\beta}{(1-\phi_{\rm PCE11})^{\gamma}}\ . \tag{10}$$

The coefficient β and the exponent γ are set to empirically-established standard values [8–10] that grant the sought numeric stability, while not impacting the physical outcome of the simulations.

B Phase-Field Model - Transport Equations

The time-evolution of the non-conserved crystallization order parameters $\psi_{\rm PCE11}$ and $\psi_{\rm PCBM}$ is governed by the stochastic Allen-Cahn equation:

$$\frac{\partial\psi_i}{\partial t} = -\frac{v_0 N_i M_i}{RT}\frac{\delta G_V}{\delta\psi_i} + \xi_i\ . \tag{11}$$

In this formula, the subscript i is again used to refer simultaneously to either of both components. The quantity M_i is a kinetic coefficient that determines the mobility of the corresponding crystal interfaces, thereby regulating the maximum speed at which crystals can grow (or dissolve). In general, $M_{\rm PCE11}$ and $M_{\rm PCBM}$ are likely to exhibit further dependencies on the volume fractions of the mixture components [8–13]. In the present study, the usually implemented composition-dependence for the mobility coefficients [10, 12] does not result in substantial qualitative changes in the simulated PCE11:PCBM blend morphologies, as compared to constant $M_{\rm PCE11}$ and $M_{\rm PCBM}$ values, so that the latter are used for simplicity.
ξ_i is a Langevin noise term that introduces stochastic fluctuations in the order parameter fields, so as to render crystal nucleation as a thermally-activated process. The noise is designed to satisfy the fluctuation-dissipation theorem [14]. Accordingly, it follows a Gaussian distribution and its standard deviation is $2v_0N_iM_i/N_A$ (where N_A is the Avogadro constant).
Furthermore, $\delta G_V/\delta\psi_i$ is a functional derivative calculated as

$$\frac{\delta G_V}{\delta\psi_i} = \frac{\partial G_V}{\partial\psi_i} - \nabla\cdot\frac{\partial G_V}{\partial(\nabla\psi_i)}\ . \tag{12}$$

Note that G_V groups all the free energy density contributions involved in the functional G, that is $G_V = G_V^{(bulk,ac)} + G_V^{(bulk,mix)} + G_V^{(grad,ac)} + G_V^{(grad,mix)} + G_V^{(ori,ac)} + G_V^{(num,mix)}$.
In contrast to the order parameters $\psi_{\rm PCE11}$ and $\psi_{\rm PCBM}$, the volume fraction $\phi_{\rm PCE11}$ is a conserved quantity. Thus, its evolution in time is dictated by the Cahn-Hilliard conservation equation, namely

$$\frac{\partial\phi_{\rm PCE11}}{\partial t} = \frac{v_0}{RT}\nabla\cdot\left[\Lambda\nabla\left(\mu_{\rm PCE11}-\mu_{\rm PCBM}\right)\right]\ , \tag{13}$$

where Λ is the Onsager coefficient that controls interdiffusion kinetics within the mixture, and $\mu_{\rm PCE11} - \mu_{\rm PCBM}$ is the so-called exchange chemical potential density, which is the thermodynamic driving force for mass transport in the present case. Considering that the PCBM volume fraction $\phi_{\rm PCBM}$ is substituted with $(1-\phi_{\rm PCE11})$ in the expression of G_V, $\mu_{\rm PCE11} - \mu_{\rm PCBM}$ is given by

$$\mu_{\rm PCE11} - \mu_{\rm PCBM} = \frac{\delta G_V}{\delta\phi_{\rm PCE11}} = \frac{\partial G_V}{\partial\phi_{\rm PCE11}} - \nabla\cdot\frac{\partial G_V}{\partial(\nabla\phi_{\rm PCE11})}\ . \tag{14}$$

The kinetics of diffusion are expected to be dependent on the concentrations of the blend components. As a consequence, so is the Onsager coefficient Λ. To model its composition-dependence, the fast mode theory [15] is employed here, leading to the following relationship:

$$\Lambda = (1-\phi_{\rm PCE11})^2\phi_{\rm PCE11}N_{\rm PCE11}D_{\rm PCE11}^{\rm (self)} + \phi_{\rm PCE11}^2(1-\phi_{\rm PCE11})N_{\rm PCBM}D_{\rm PCBM}^{\rm (self)}\ . \tag{15}$$

In this latter equation, $D^{(\mathrm{self})}_{\mathrm{PCE11}}$ and $D^{(\mathrm{self})}_{\mathrm{PCBM}}$ stand for the self-diffusion coefficients of PCE11 and PCBM, respectively. These quantities are also varying with the mixture composition. A logarithmic mean is utilized in this study to interpolate their values between the infinitesimal dilution and the pure component limits:

$$\begin{cases} D^{(\mathrm{self})}_{\mathrm{PCE11}} = \left(D^{(\mathrm{self})}_{\mathrm{PCE11}|\phi\to 0}\right)^{(1-\phi_{\mathrm{PCE11}})} \left(D^{(\mathrm{self})}_{\mathrm{PCE11}|\phi\to 1}\right)^{\phi_{\mathrm{PCE11}}} , \\ D^{(\mathrm{self})}_{\mathrm{PCBM}} = \left(D^{(\mathrm{self})}_{\mathrm{PCBM}|\phi\to 0}\right)^{\phi_{\mathrm{PCE11}}} \left(D^{(\mathrm{self})}_{\mathrm{PCBM}|\phi\to 1}\right)^{(1-\phi_{\mathrm{PCE11}})} . \end{cases} \tag{16}$$

$D^{(\mathrm{self})}_{\mathrm{PCE11}|\phi\to 0}$ and $D^{(\mathrm{self})}_{\mathrm{PCE11}|\phi\to 1}$ label the self-diffusion coefficients in these extremal cases for PCE11. Analogously, $D^{(\mathrm{self})}_{\mathrm{PCBM}|\phi\to 0}$ and $D^{(\mathrm{self})}_{\mathrm{PCBM}|\phi\to 1}$ are the self-diffusion coefficients for PCBM, first, infinitely diluted in PCE11, and, second, in the pure material.
Finally, it can be remarked that the material diffusivities are anticipated to be drastically reduced within crystals. Therefore, a penalty is applied to the self-diffusion coefficients $D^{(\mathrm{self})}_{\mathrm{PCE11}}$ and $D^{(\mathrm{self})}_{\mathrm{PCBM}}$ depending on the values of the order parameters ψ_{PCE11} and ψ_{PCBM}, as further detailed by Ronsin and Harting [10]. In the tables of the upcoming section, k_d denotes the reduction factor that multiplies $D^{(\mathrm{self})}_{\mathrm{PCE11}}$ and $D^{(\mathrm{self})}_{\mathrm{PCBM}}$ in fully crystalline domains (i.e. where $\psi_{\mathrm{PCE11}} = 1$ or $\psi_{\mathrm{PCBM}} = 1$).

C Phase-Field Model - Parameter Tables

All model parameter values used for the Phase-Field simulations of this work are reproduced in this section. First, the material parameters gathered in Tab. 1 are discussed. The density (1600 kg/m^3) and the molar mass (0.91 kg/mol) of the PCBM small molecule are well-known and usually already provided by the material producers. For the PCE11 polymer, these properties are less straightforward to assess. Here, the PCE11 density is estimated at 1100 kg/m^3, which is a standard first-order approximation for polymers. The molar mass is set according to the value reported by Levitsky et al. [16, 17] for the batch employed in the SEM experiments (83 kg/mol). The molar volumes of both species are then calculated from the ratio of their respective molar masses to their densities. Next, the molar volume of the Flory-Huggins lattice elements v_0 is taken equal to that of the PCBM, so that the small molecule size in terms of Flory-Huggins lattice elements is N_{PCBM} = 1. Consequently, the size of the PCE11 polymer is N_{PCE11} = 132.667. Using the aforementioned densities and the overall PCE11 mass fraction $m_{\%,\mathrm{PCE11}}$ in the active layers fabricated by Levitsky et al. [17] ($m_{\%,\mathrm{PCE11}}$ = 0.45), the total volume fractions of PCE11 ($\phi_{0,\mathrm{PCE11}}$) and PCBM ($\phi_{0,\mathrm{PCBM}}$) are calculated with the following relationships:

$$\phi_{0,\mathrm{PCE11}} = \frac{m_{\%,\mathrm{PCE11}}}{m_{\%,\mathrm{PCE11}} + \frac{\rho_{\mathrm{PCE11}}}{\rho_{\mathrm{PCBM}}}(1 - m_{\%,\mathrm{PCE11}})} \text{ , and } \phi_{0,\mathrm{PCBM}} = 1 - \phi_{0,\mathrm{PCE11}} . \tag{17}$$

These volume fractions are the same for all simulations presented in the main body of the article ($\phi_{0,\mathrm{PCE11}}$ = 0.55, $\phi_{0,\mathrm{PCBM}}$ = 0.45), although variations are performed in SI-D, SI-E, and SI-F to examine the impact of the blend ratio on morphology. In the simulations accounting for the effect of PCE11 crystallites, the initial crystallinity of PCE11 ($\psi_{0,\mathrm{PCE11}}$) amounts to around 33%, with 948 isotropic crystal seeds with a diameter of 16 nm that are placed randomly within the 1024 nm^2 2D simulation box.

Parameter	Symbol	Value	Unit
PCE11 density	ρ_{PCE11}	1100	kg/m^3
PCBM density	ρ_{PCBM}	1600	kg/m^3
PCE11 Flory-Huggins size coefficent	N_{PCE11}	132.667	-
PCBM Flory-Huggins size coefficent	N_{PCBM}	1	-
Flory-Huggins lattice element molar volume	v_0	5.6875$\times$10^{-4}	m^3/mol
PCE11 total volume fraction	$\phi_{0,\mathrm{PCE11}}$	0.55 *	-
PCBM total volume fraction	$\phi_{0,\mathrm{PCBM}}$	0.45 *	-
Initial PCE11 crystallinity	$\psi_{0,\mathrm{PCE11}}$	(0) 0.33	-
Initial number of PCE11 crystallites	-	(0) 948 *	-
PCE11 crystallite diameter	-	16$\times$10^{-9}	m

Table 1: Material parameters used in the thermal annealing simulations of the PCE11:PCBM mixture. Parameter values marked with an asterisk are susceptible to be varied in the following sections (SI-D, SI-E, SI-F, and SI-G). Moreover, values that differ in simulation cases without initial PCE11 crystallites are reported within parentheses.

Furthermore, the values of the thermodynamic model parameters are listed in Tab. 2. The simulated system temperature T agrees with the annealing temperature at which the morphology evolution monitoring is carried out by Levitsky et al. [17] (130 °C). The melting temperatures of the pure PCE11 and pure PCBM components are readily available in various publications that present DSC data [16, 18–21], including the phase diagram assessment of Levitsky et al. [16]. The latent heat of fusion of PCBM is also specified in multiple reports with values generally ranging between 5000 J/kg and 25000 J/kg [16, 19, 22–25]. In the present work, L_{PCBM} = 15000 J/kg is found to yield the best agreement when fitting the measured PCBM melting point depression. The equilibrium heat of fusion of PCE11 is evaluated at 50350 $\pm$ 4150 J/kg by Perea et al. [18]. This value indeed allows to reproduce accurately the melting point depression in the vicinity of the melting temperature of pure PCE11. However, it leads to an overestimation of the eutectic temperature in the blend. Therefore, L_{PCE11} = 30000 J/kg is used in this study, which is in line with the DSC scans of Levitsky et al. [16] and permits to recover the eutectic point while still giving a reasonable estimate of the PCE11 melting point depression near $T_{m,\mathrm{PCE11}}$. Further dependencies on composition may be considered for the fusion enthalpy parameters to refine the prediction of the melting point depression. Nevertheless, a high precision on the assessment of L_{PCE11} is not critical for the present endeavour, especially considering that the main effect observed for PCE11 crystallites on blend morphology is due to geometric and kinetic factors. The molar enthalpies of fusion $\Delta h_{\mathrm{PCE11}}$ and Δh_{PCBM} are then obtained by multiplying the latent heats L_{PCE11} and L_{PCBM} in J/kg units by the corresponding molar masses of the species.
The value of the Flory-Huggins interaction parameter follows from the melting point depression fit described in the main text. According to Perea et al. [18], its entropic part A is regarded as negligible for polymer:small molecule systems. The remaining B/T contribution of Eq. 7 is further substituted for $\chi^{(aa)}$ in the melting point depression equation with $T = T_d$ (T_d referring to the depressed melting temperature in the blend). Knowing the depressed melting temperatures for different blend ratios as well as the heats of fusion, the coefficient B is the only degree of freedom left, which can be calibrated in order to match the calculated liquidi of PCBM and PCE11 to the experimental phase diagram. Here, this results in B = 509.76 K, which yields $\chi^{(aa)}$ values in line with the work of Perea et al. [18]. The simulations relying on the present thermodynamic parameter set are not sensitive to the exact values of the crystal-amorphous interaction parameters $\chi^{(ca)}$ and $\chi^{(ac)}$, as long as they are high enough to provide pure PCE11 and PCBM crystal phases. The associated scaling coefficients C_{PCE11} = 9.6390$\times$10^{-3} and C_{PCBM} = 1.0990 are used in Eq. 8 to fulfill this purpose. In addition, the crystal-crystal interaction parameter $\chi^{(cc)}$ is mainly useful when it is sought to model co-crystals. Thus, its value is set to 0.

Parameter	Symbol	Value	Unit
Temperature	T	403	K
PCE11 melting temperature	$T_{m,\mathrm{PCE11}}$	555	K
PCBM melting temperature	$T_{m,\mathrm{PCBM}}$	558	K
PCE11 latent heat of fusion	L_{PCE11}	30000	J/kg
PCBM latent heat of fusion	L_{PCBM}	15000	J/kg
PCE11 molar enthalpy of fusion	$\Delta h_{\mathrm{PCE11}}$	2490000	J/mol
PCBM molar enthalpy of fusion	Δh_{PCBM}	13650	J/mol
PCE11 energy barrier coefficent	W_{PCE11}	41081	J/kg
PCBM energy barrier coefficent	W_{PCBM}	25000 *	J/kg
Flory-Huggins interaction parameter (amorphous-amorphous)	$\chi^{(aa)}$	1.2649	-
Entropy coefficient of the Flory-Huggins interaction parameter	A	0	-
Enthalpy coefficient of the Flory-Huggins interaction parameter	B	509.76	K
Interaction parameter for crystalline PCE11 and amorphous PCBM	$\chi^{(ca)}$	7.1633	-
Proportionality coefficient of the PCE11 crystal interaction parameter	C_{PCE11}	9.6390×10^{-3}	-
Interaction parameter for amorphous PCE11 and crystalline PCBM	$\chi^{(ac)}$	4.4771 *	-
Proportionality coefficient of the PCBM crystal interaction parameter	C_{PCBM}	1.0990 *	-
Crystal-crystal interaction parameter	$\chi^{(cc)}$	0	-

Table 2: Thermodynamic parameters used in the thermal annealing simulations of the PCE11:PCBM mixture. Parameter values marked with an asterisk are susceptible to be varied in the following sections (SI-D, SI-E, SI-F, and SI-G).

The value of the energy barrier coefficients for the PCE11 polymer (W_{PCE11}) and the PCBM small molecule (W_{PCBM}) are selected to respect the nucleation and growth limit criterion inherent to the employed crystallization free energy form with the polynomials $p(\psi_i)$ and $q(\psi_i)$ [13, 26]. What is more, these energy barrier coefficients determine the surface tension of crystals together with the surface tension coefficients $\varepsilon_{\mathrm{PCE11}}$ and $\varepsilon_{\mathrm{PCBM}}$ [1]. As a consequence, they influence the minimum size for which crystal nuclei are stable. Therefore, W_{PCE11} and $\varepsilon_{\mathrm{PCE11}}$ are also chosen low enough to maintain a PCE11 critical radius below 8 nm, so as to ensure the stability of the initial PCE11 crystallites. Similarly, the value of W_{PCBM} is adjusted in combination with that of $\varepsilon_{\mathrm{PCBM}}$ to reproduce the desired growth-dominated PCBM crystallization behavior (SI-E details further the impact of surface tension on the nucleation density, the growth pattern, and the overall blend morphology). Note that the balance between both parameters matters as well, as it dictates the thickness of the diffuse crystal interfaces, which is required to span over at least four mesh points to grant sufficient numerical convergence properties [1].
All the surface tension coefficients are tabulated in Tab. 3 along with the crystal impingement energy coefficients. The values of the latter (α_{PCE11} and α_{PCBM}) are adapted from previous studies [12, 13] to prevent the interpenetration of distinct crystals with different orientations. The values of the surface tension coefficients for interfaces between phases of different compositions (i.e., κ_{PCE11} and κ_{PCBM}) are taken over as well from earlier investigations [12, 13]. It is found that, without further fine-tuning, the implied length and timescales for the initial amorphous demixing (which are conjointly influenced by other parameters such as the blend ratio, or the Flory-Huggins interaction parameter [27]) agree with the observations from SEM experiments [17].

Parameter	Symbol	Value	Unit
PCE11 crystal surface tension coefficient	$\varepsilon^2_{\mathrm{PCE11}}$	1×10^{-10}	J/m
PCBM crystal surface tension coefficient	$\varepsilon^2_{\mathrm{PCBM}}$	4.84×10^{-10} *	J/m
PCE11 impingement energy coefficient	α_{PCE11}	16.32×10^{7}	J/m^3
PCBM impingement energy coefficient	α_{PCBM}	16.32×10^{7}	J/m^3
General PCE11 domain surface tension coefficient	κ_{PCE11}	1×10^{-10}	J/m
General PCBM domain surface tension coefficient	κ_{PCBM}	1×10^{-10}	J/m

Table 3: Surface tension and crystal impingement parameters used in the thermal annealing simulations of the PCE11:PCBM mixture. Parameter values marked with an asterisk are susceptible to be varied in the following sections (SI-D, SI-E, SI-F, and SI-G).

Tab. 4 reproduces the kinetic parameters used to simulate the PCE11:PCBM system under thermal annealing. The mobility coefficient for PCE11 crystals M_{PCE11} is set relatively low, so that both PCE11 crystal nucleation and growth are hindered during the annealing process, as expected from the experimental characterizations [17]. Conversely, the parameter M_{PCBM} is calibrated to obtain PCBM crystallization on timescales that match with the morphology evolution evidenced by Levitsky et al. [17]. The values of the self-diffusion coefficients in the pure materials stem from a prior Phase-Field analysis of the P3HT:PCBM bulk heterojunction by Ronsin and Harting [12]. With the modeling assumptions specified in SI-B to render the composition-dependence of the material diffusivities, these parameter values lead to initial amorphous spinodal decomposition timescales in agreement with the process kinetics revealed by the SEM measurements [17].
The penalty coefficient k_d, which slows down diffusion inside crystals, must provide a diffusion coefficient reduction by several orders of magnitude to render the physical material transport behavior within these regions. However, k_d cannot be taken arbitrarily low in simulations with PCE11 crystallites, as it also affects their dissolution rate. More specifically, when the diffusion in PCE11 crystal regions becomes too low (due to the penalty factor) as compared to the growth rate of PCBM crystals (meaning that, in terms of model parameters, the balance between $k_d\Lambda$, when $\phi_{\mathrm{PCE11}} \to 1$, and M_{PCBM} is too low), PCE11 crystallite residuals may be retained within PCBM crystals as a result of the kinetically impaired PCE11 dissolution (see SI-F). Without the initial PCE11 crystallites, the value of the parameter k_d is less impactful for the final morphology, as long as it is low enough.

**Parameter**	**Symbol**	**Value**	**Unit**
PCE11 crystal mobility coefficient	M_{PCE11}	1×10^{-10}	s^{-1}
PCBM crystal mobility coefficient	M_{PCBM}	(1×10^{-1}) 2.5×10^{-2} *	s^{-1}
PCE11 self-diffusion coefficient in pure PCE11	$D^{(\mathrm{self})}_{\mathrm{PCE11}\vert\phi\to1}$	1×10^{-16}	m^2/s
PCE11 self-diffusion coefficient in pure PCBM	$D^{(\mathrm{self})}_{\mathrm{PCE11}\vert\phi\to0}$	5×10^{-16}	m^2/s
PCBM self-diffusion coefficient in pure PCE11	$D^{(\mathrm{self})}_{\mathrm{PCBM}\vert\phi\to0}$	4×10^{-15}	m^2/s
PCBM self-diffusion coefficient in pure PCBM	$D^{(\mathrm{self})}_{\mathrm{PCBM}\vert\phi\to1}$	1×10^{-13}	m^2/s
Diffusion penalty factor in crystalline domains	k_d	(1×10^{-6}) 2.5×10^{-5} *	-

Table 4: Kinetic parameters used in the thermal annealing simulations of the PCE11:PCBM mixture. Parameter values marked with an asterisk are susceptible to be varied in the following sections (SI-D, SI-E, SI-F, and SI-G). Moreover, values that differ in simulation cases without initial PCE11 crystallites are reported within parentheses.

Finally, Tab. 5 contains the numeric parameters that are relevant for the simulation of the studied material mixture. The values of the regularization parameters β and γ are established empirically [10], such as to prevent numeric instabilities when phases tend to become perfectly pure (i.e. $\phi_{\mathrm{PCE11}} \to 0$ or $\phi_{\mathrm{PCE11}} \to 1$), as already mentioned in SI-A. The two-dimensional domain in which the field variables are monitored in the simulations is a 1024 nm by 1024 nm square box with periodic boundary conditions. The size of the 1048576 discrete mesh elements that constitute the domain is 1 nm by 1 nm. To optimize computational effort, an adaptive time-stepping scheme is utilized to calculate the system's evolution in time with the largest possible timestep which still provides numerical convergence. More details about the implementation of the Phase-Field model and the resolution schemes for the discretized transport equations can be found in the computational framework description by Ronsin and Harting [10].

**Parameter**	**Symbol**	**Value**	**Unit**
Regularization coefficient	β	1×10^{-5}	J/m^3
Regularization exponent	γ	1	-
Total simulation box size	-	1024 *	nm^2
Mesh element size (resolution)	-	1	nm^2
Timestep	-	Adaptive	s

Table 5: Numeric parameters used in the thermal annealing simulations of the PCE11:PCBM mixture. Parameter values marked with an asterisk are susceptible to be varied in the following sections (SI-D, SI-E, SI-F, and SI-G).

D Effect of Blend Ratio on Spinodal Decomposition Morphology

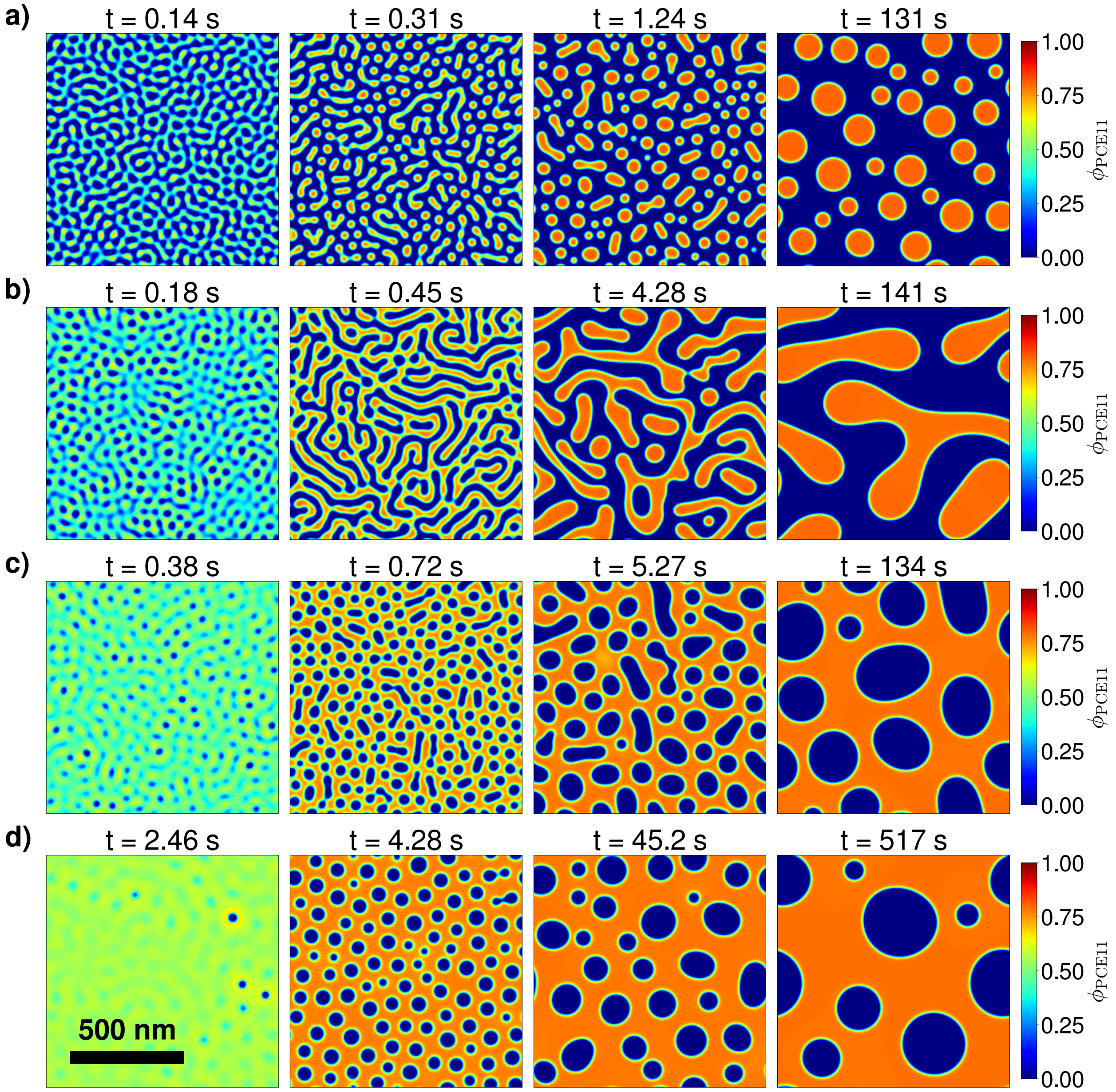

Figure 1: Simulations of PCE11:PCBM mixtures undergoing amorphous spinodal decomposition at T = 130 °C. The morphology evolution is strongly dependent on the blend ratio, as shown here for a) $\phi_{0,\mathrm{PCE11}}$ = 0.25, b) $\phi_{0,\mathrm{PCE11}}$ = 0.35, c) $\phi_{0,\mathrm{PCE11}}$ = 0.45, and d) $\phi_{0,\mathrm{PCE11}}$ = 0.55. The calculations are performed with the Phase-Field model described in SI-A and SI-B, and with the parameters reported in SI-C. Note that crystallization is deactivated (i.e. ψ_{PCE11} = ψ_{PCBM} = 0), so that only diffusive mass transport governed by Cahn-Hilliard kinetics shapes the observed phase transformations. Since the Langevin term of the Allen-Cahn equation is not effective in this particular case (see SI-B), an initial mass-conservative noise is applied to the volume fraction field ϕ_{PCE11} in order to reproduce the effect of thermal fluctuations on the system and allow for the demixing to proceed.

E Effect of Surface Tension on Crystal Size and Density

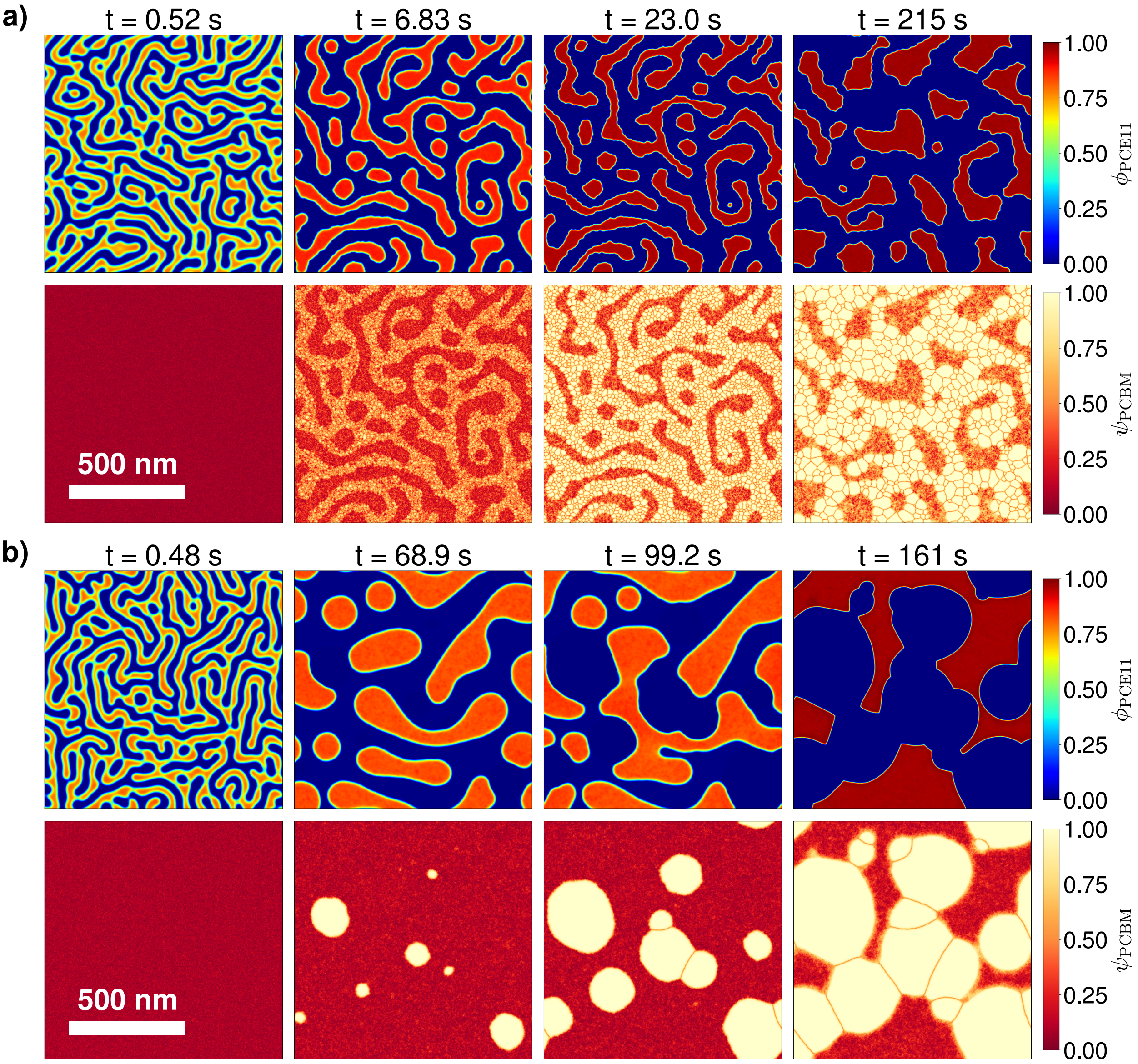

Figure 2: Simulations of PCE11:PCBM mixtures with different PCBM crystal surface tension parameters at T = 130 °C. a) Low surface tension with $\varepsilon^2_{\text{PCBM}}$ = 1×10^{-10} J/m, W_{PCBM} = 20833 J/kg, and $\chi^{(ac)}$ = 2.6863, that is C_{PCBM} = 0.6594. b) High surface tension with $\varepsilon^2_{\text{PCBM}}$ = 4.84×10^{-10} J/m, W_{PCBM} = 25000 J/kg, and $\chi^{(ac)}$ = 4.4771, that is C_{PCBM} = 1.0990. All other Phase-Field model parameters are identical in both simulation cases, with an overall blend ratio corresponding to $\phi_{0,\text{PCE11}}$ = 0.35. The remaining parameter values are those specified in the tables of SI-C. Initial PCE11 crystallites are not placed in the present mixtures. For each simulation, the upper and lower rows respectively show the evolution of the PCE11 volume fraction (ϕ_{PCE11}) and the PCBM order parameter (ψ_{PCBM}) fields.

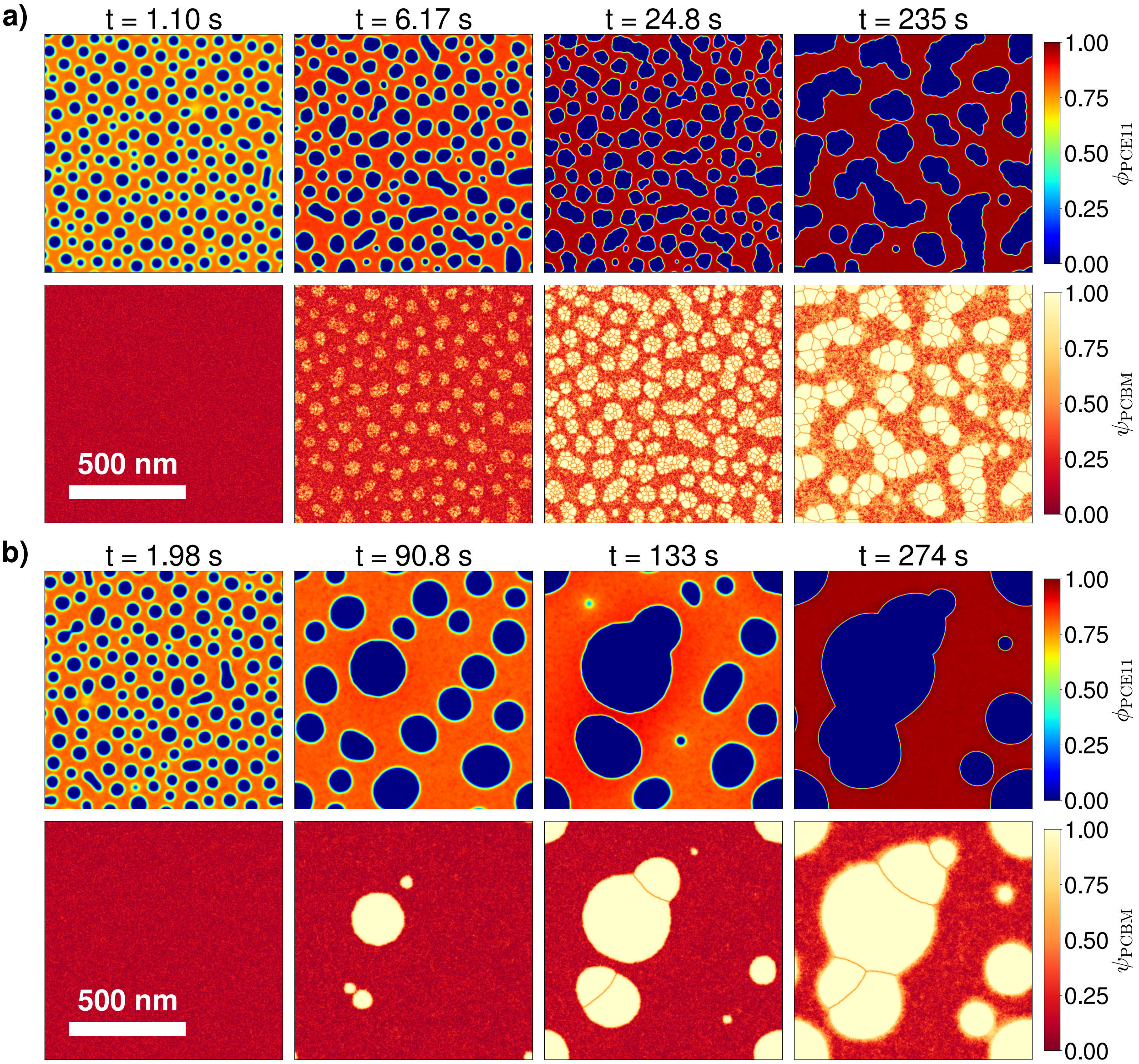

Figure 3: Simulations of PCE11:PCBM mixtures with different PCBM crystal surface tension parameters at T = 130 °C. a) Low surface tension with $\varepsilon^2_{\mathrm{PCBM}}$ = 1×10^{-10} J/m, W_{PCBM} = 20833 J/kg, and $\chi^{(ac)}$ = 2.6863, that is C_{PCBM} = 0.6594. b) High surface tension with $\varepsilon^2_{\mathrm{PCBM}}$ = 4.84×10^{-10} J/m, W_{PCBM} = 25000 J/kg, and $\chi^{(ac)}$ = 4.4771, that is C_{PCBM} = 1.0990. All other Phase-Field model parameters are identical in both simulation cases, with an overall blend ratio corresponding to $\phi_{0,\mathrm{PCE11}}$ = 0.55. The remaining parameter values are those specified in the tables of SI-C. Initial PCE11 crystallites are not placed in the present mixtures. For each simulation, the upper and lower rows respectively show the evolution of the PCE11 volume fraction (ϕ_{PCE11}) and the PCBM order parameter (ψ_{PCBM}) fields.

F Effect of Diffusion-Limited Growth on Crystal Arrangement

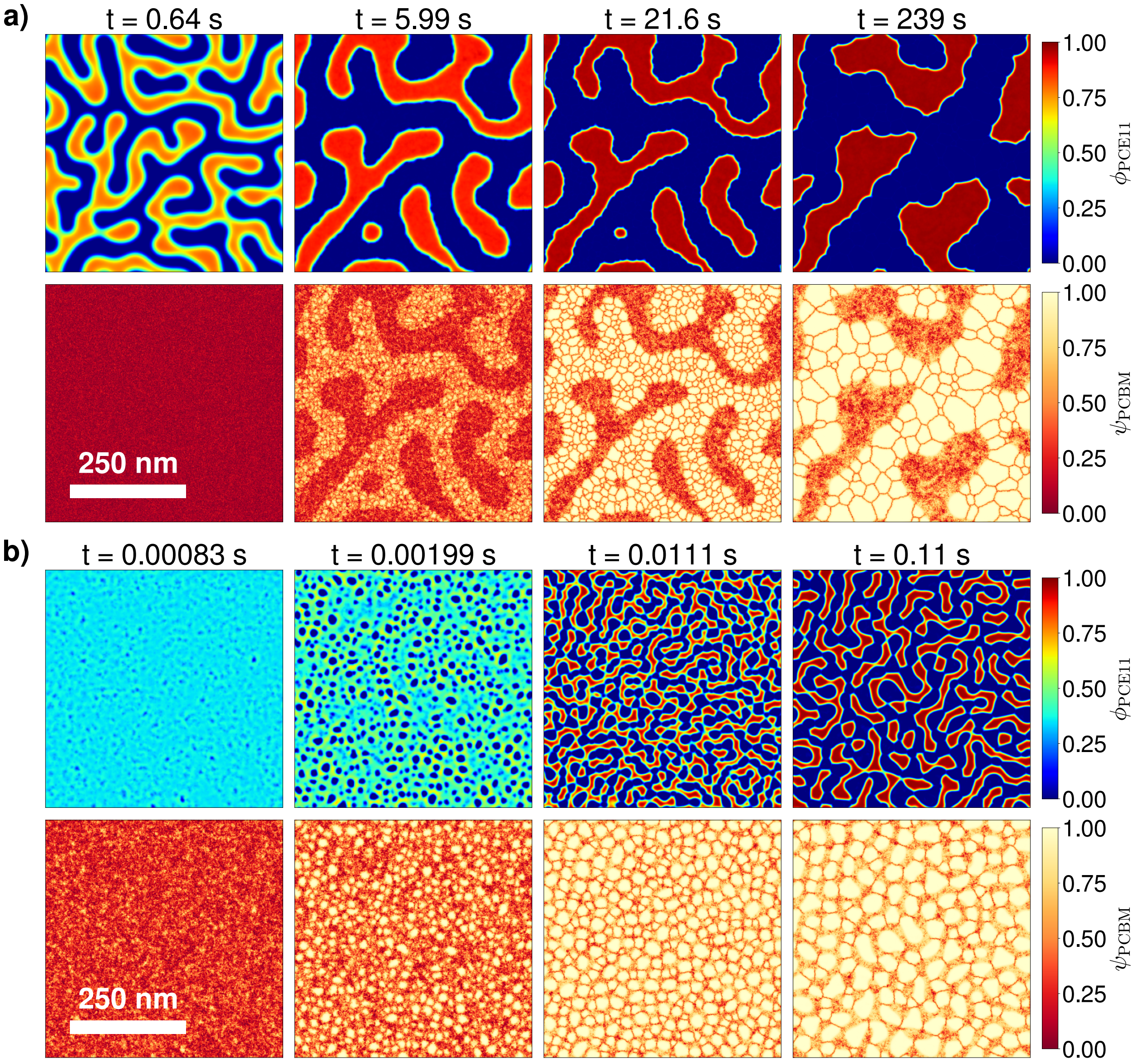

Figure 4: Simulations of PCE11:PCBM mixtures with different PCBM crystal growth kinetics at T = 130 °C. a) Non-diffusion-limited system with M_{PCBM} = 1×10^{-1} s^{-1}. b) Diffusion-limited system with M_{PCBM} = 1000 s^{-1}. All other Phase-Field model parameters are identical in both simulation cases, with an overall blend ratio corresponding to $\phi_{0,\mathrm{PCE11}}$ = 0.35. The surface tension of the PCBM crystals is adjusted to be relatively low (i.e. $\varepsilon^2_{\mathrm{PCBM}}$ = 1×10^{-10} J/m, W_{PCBM} = 20833 J/kg, and $\chi^{(ac)}$ = 2.6863), so that the crystallization process is globally nucleation-dominated. The simulation domains are 512 nm^2 square boxes with periodic boundary conditions. The remaining parameter values are those specified in the tables of SI-C. Initial PCE11 crystallites are not placed in the present mixtures. For each simulation, the upper and lower rows respectively show the evolution of the PCE11 volume fraction (ϕ_{PCE11}) and the PCBM order parameter (ψ_{PCBM}) fields.

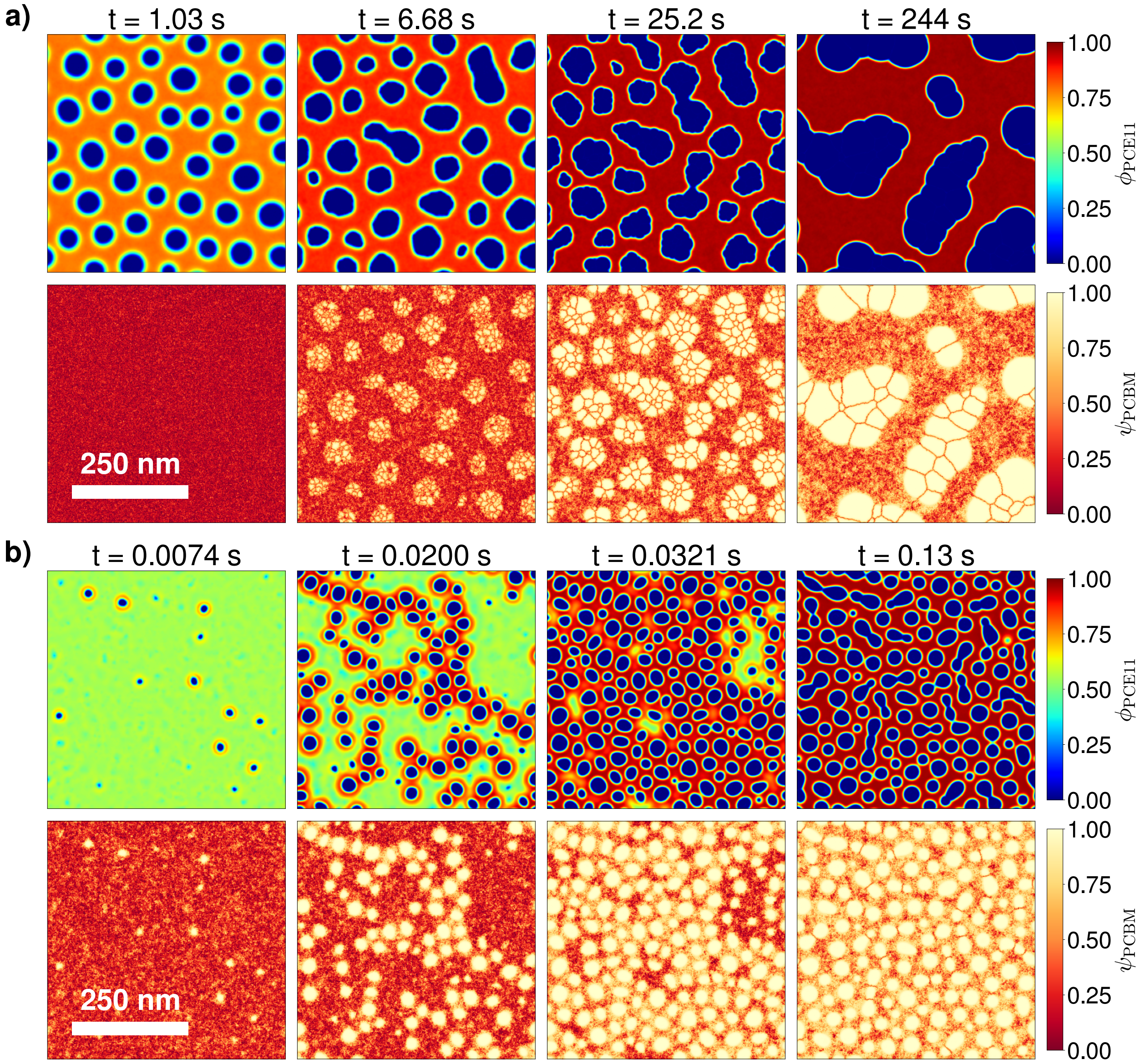

Figure 5: Simulations of PCE11:PCBM mixtures with different PCBM crystal growth kinetics at T = 130 °C. a) Non-diffusion-limited system with M_{PCBM} = 1×10^{-1} s^{-1}. b) Diffusion-limited system with M_{PCBM} = 1000 s^{-1}. All other Phase-Field model parameters are identical in both simulation cases, with an overall blend ratio corresponding to $\phi_{0,\mathrm{PCE11}}$ = 0.55. The surface tension of the PCBM crystals is adjusted to be relatively low (i.e. $\varepsilon^2_{\mathrm{PCBM}}$ = 1×10^{-10} J/m, W_{PCBM} = 20833 J/kg, and $\chi^{(ac)}$ = 2.6863), so that the crystallization process is globally nucleation-dominated. The simulation domains are 512 nm^2 square boxes with periodic boundary conditions. The remaining parameter values are those specified in the tables of SI-C. Initial PCE11 crystallites are not placed in the present mixtures. For each simulation, the upper and lower rows respectively show the evolution of the PCE11 volume fraction (ϕ_{PCE11}) and the PCBM order parameter (ψ_{PCBM}) fields.

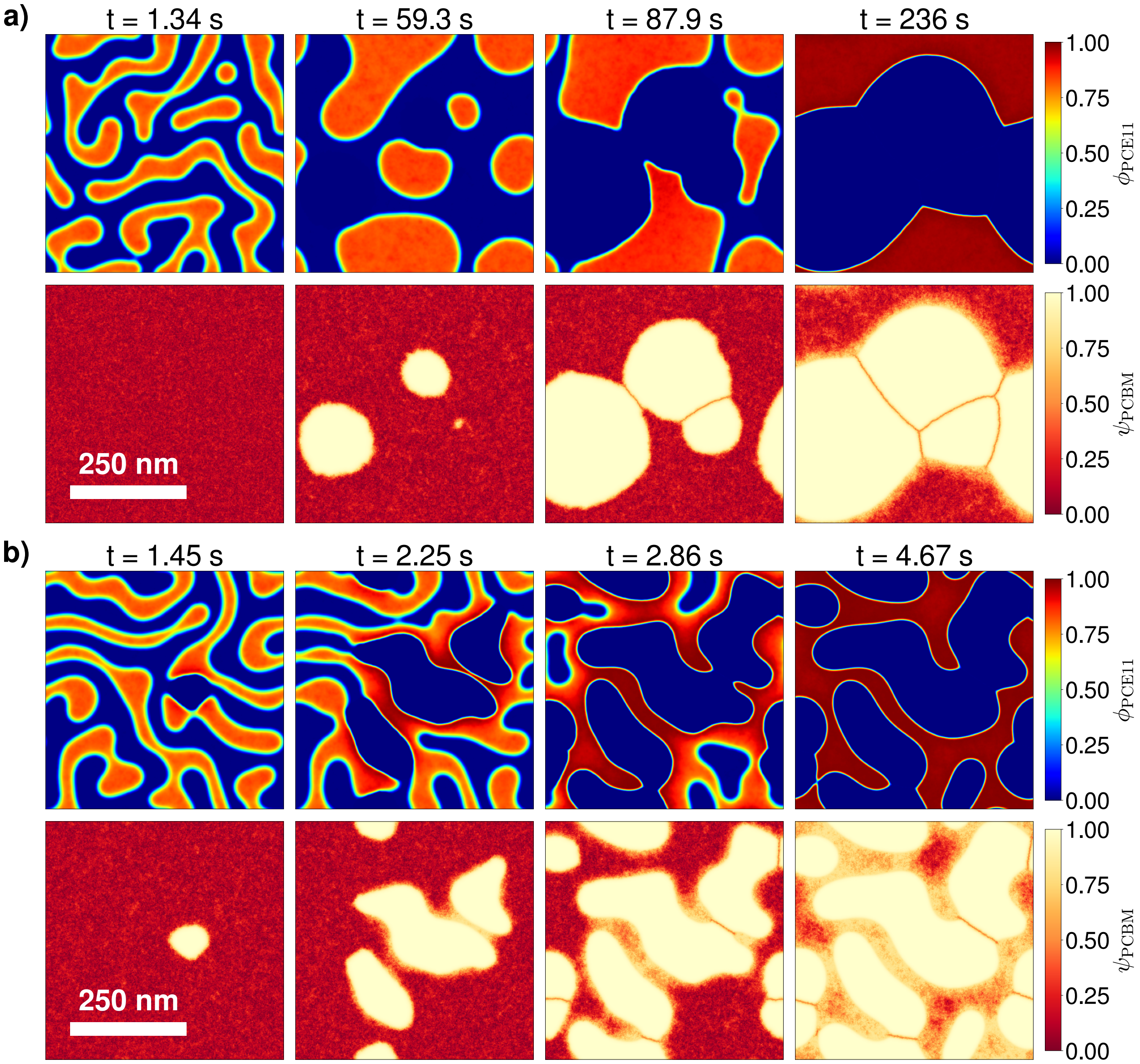

Figure 6: Simulations of PCE11:PCBM mixtures with different PCBM crystal growth kinetics at T = 130 °C. a) Non-diffusion-limited system with M_{PCBM} = 1×10^{-1} s^{-1}. b) Diffusion-limited system with M_{PCBM} = 10 s^{-1}. All other Phase-Field model parameters are identical in both simulation cases, with an overall blend ratio corresponding to $\phi_{0,\mathrm{PCE11}}$ = 0.35. The surface tension of the PCBM crystals is adjusted to be relatively high (i.e. $\varepsilon^2_{\mathrm{PCBM}}$ = 4.84×10^{-10} J/m, W_{PCBM} = 25000 J/kg, and $\chi^{(ac)}$ = 4.4771), so that the crystallization process is globally growth-dominated. The simulation domains are 512 nm^2 square boxes with periodic boundary conditions. The remaining parameter values are those specified in the tables of SI-C. Initial PCE11 crystallites are not placed in the present mixtures. For each simulation, the upper and lower rows respectively show the evolution of the PCE11 volume fraction (ϕ_{PCE11}) and the PCBM order parameter (ψ_{PCBM}) fields.

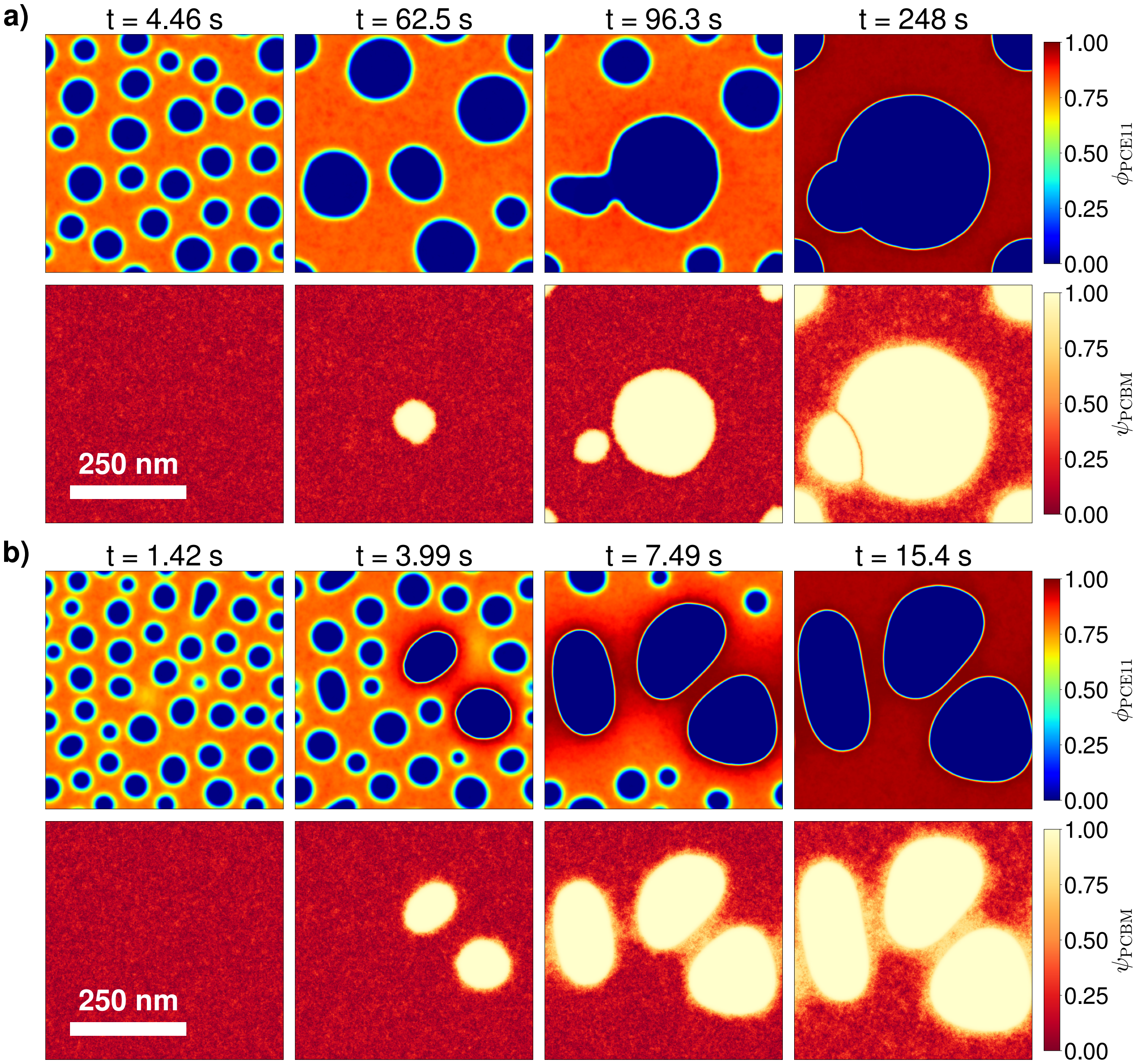

Figure 7: Simulations of PCE11:PCBM mixtures with different PCBM crystal growth kinetics at T = 130 °C. a) Non-diffusion-limited system with M_{PCBM} = 1×10^{-1} s^{-1}. b) Diffusion-limited system with M_{PCBM} = 10 s^{-1}. All other Phase-Field model parameters are identical in both simulation cases, with an overall blend ratio corresponding to $\phi_{0,\mathrm{PCE11}}$ = 0.55. The surface tension of the PCBM crystals is adjusted to be relatively high (i.e. $\varepsilon^2_{\mathrm{PCBM}}$ = 4.84×10^{-10} J/m, W_{PCBM} = 25000 J/kg, and $\chi^{(ac)}$ = 4.4771), so that the crystallization process is globally growth-dominated. The simulation domains are 512 nm^2 square boxes with periodic boundary conditions. The remaining parameter values are those specified in the tables of SI-C. Initial PCE11 crystallites are not placed in the present mixtures. For each simulation, the upper and lower rows respectively show the evolution of the PCE11 volume fraction (ϕ_{PCE11}) and the PCBM order parameter (ψ_{PCBM}) fields.

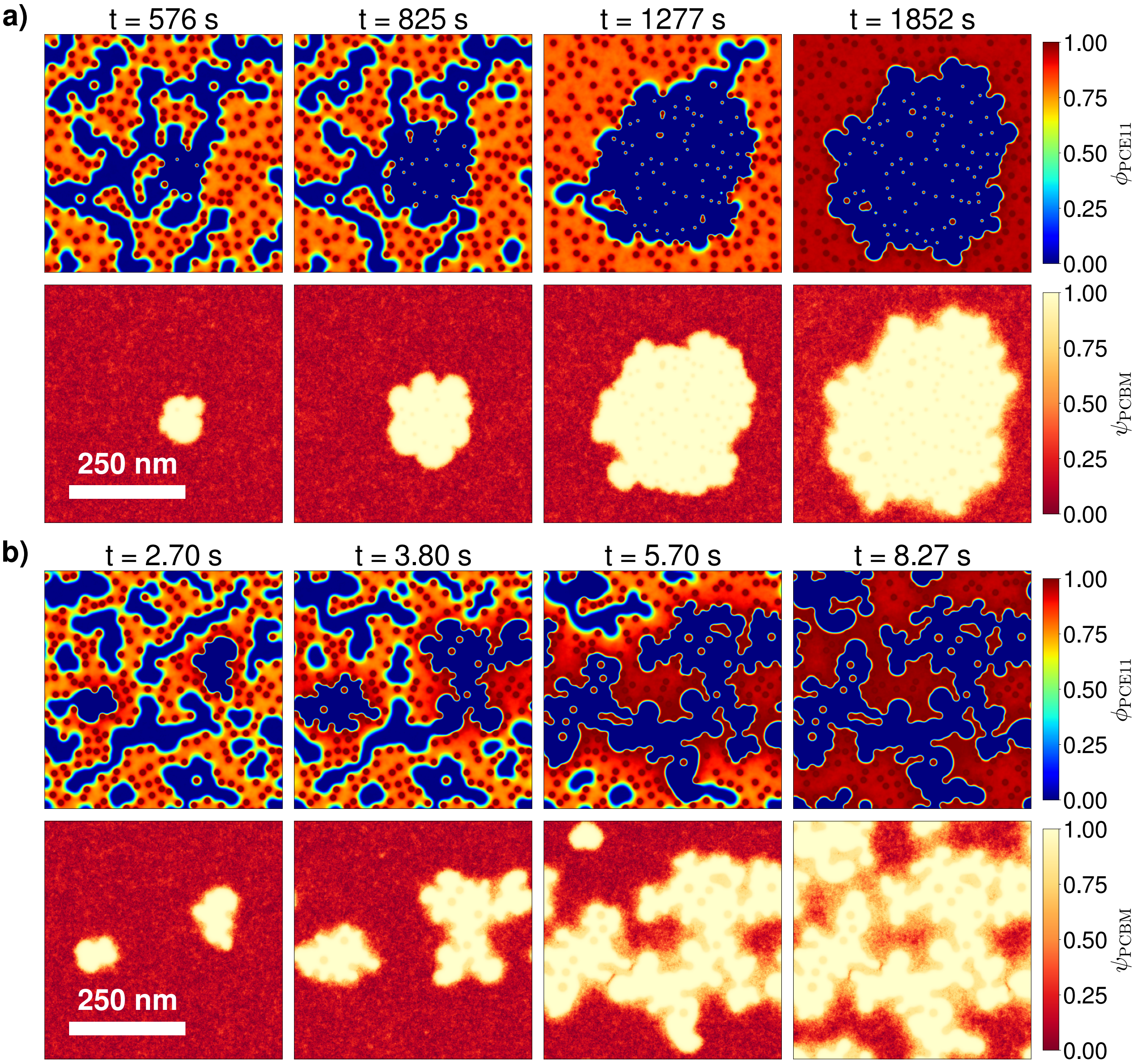

Figure 8: Simulations of PCE11:PCBM mixtures at T = 130 °C with randomly placed initial PCE11 crystallites and different PCBM crystal growth kinetics. a) Non-diffusion-limited system in the amorphous phase with M_{PCBM} = 1×10^{-2} s^{-1}. Nevertheless, the diffusion penalty factor k_d = 1×10^{-6} hinders PCE11 crystallite dissolution at the PCBM growth front, leading to PCE11 crystallite residuals that remain trapped within the PCBM crystal. b) Diffusion-limited system with M_{PCBM} = 10 s^{-1}. Here, k_d is also set to 1×10^{-6}. Its exact value is however not critical in this particular case, as the anisotropic, branching crystalline PCBM domain shapes with incorporated PCE11 crystallites are dictated by the PCBM diffusion through the PCE11-rich amorphous phase, which is slow as compared to the relatively fast growth speed of PCBM crystals. All other Phase-Field model parameters are identical in both simulation cases, with an overall blend ratio corresponding to $\phi_{0,\mathrm{PCE11}}$ = 0.55. The surface tension of the PCBM crystals is adjusted to be relatively high (i.e. $\varepsilon^2_{\mathrm{PCBM}}$ = 4.84×10^{-10} J/m, W_{PCBM} = 25000 J/kg, and $\chi^{(ac)}$ = 4.4771), so that the crystallization process is globally growth-dominated. The simulation domains are 512 nm^2 square boxes with periodic boundary conditions. The remaining parameter values are those specified in the tables of SI-C. For each simulation, the upper and lower rows respectively show the evolution of the PCE11 volume fraction (ϕ_{PCE11}) and the PCBM order parameter (ψ_{PCBM}) fields.

G Effect of PCE11 Crystallites - Additional Simulations

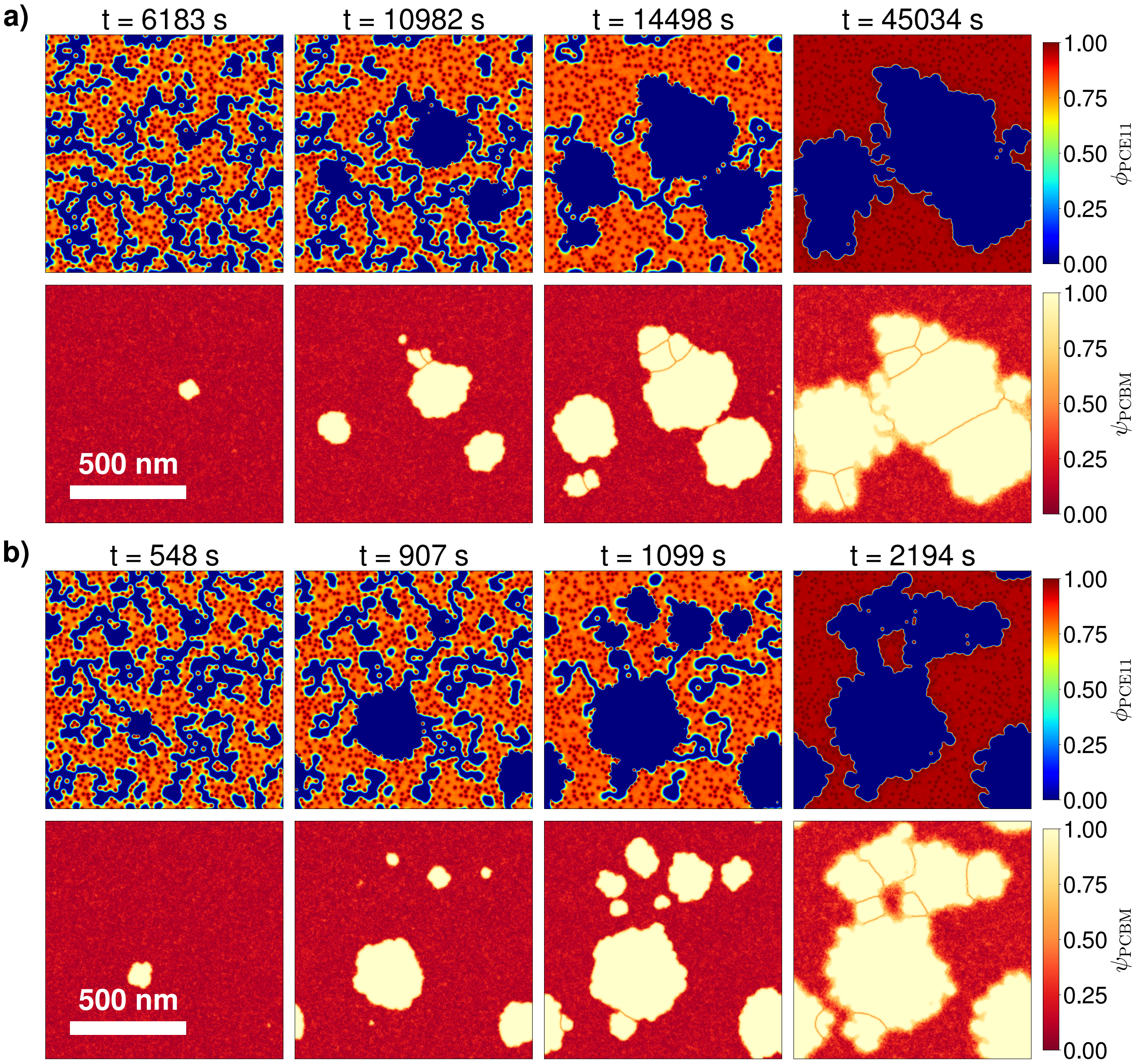

Figure 9: Simulations of PCE11:PCBM mixtures at T = 130 °C with randomly placed initial PCE11 crystallites and a delayed PCBM crystallization process (as compared to the case presented in the main body of the article). In a), the PCBM crystallization kinetics experience a relatively strong slowdown with $M_{\rm PCBM}$ = $1{\times}10^{-3}$ s^{-1}. The diffusivity reduction coefficient in crystalline domains k_d is set here to $1{\times}10^{-6}$. In b), the PCBM crystallization kinetics experience a more moderate slowdown with $M_{\rm PCBM}$ = $1.5{\times}10^{-2}$ s^{-1}. The diffusivity reduction coefficient in crystalline domains k_d is set here to $1.5{\times}10^{-5}$. All unspecified model parameters are those reported in the tables of SI-C. Due to the quench of the amorphous phase separation pattern by the PCE11 crystallites, the demixing-assisted PCBM crystallization results in crystalline domains with similar geometries, crystal sizes, and grain numbers, independently of the timescale shift induced by the variation of $M_{\rm PCBM}$. For each simulation, the upper and lower rows respectively show the evolution of the PCE11 volume fraction ($\phi_{\rm PCE11}$) and the PCBM order parameter ($\psi_{\rm PCBM}$) fields.

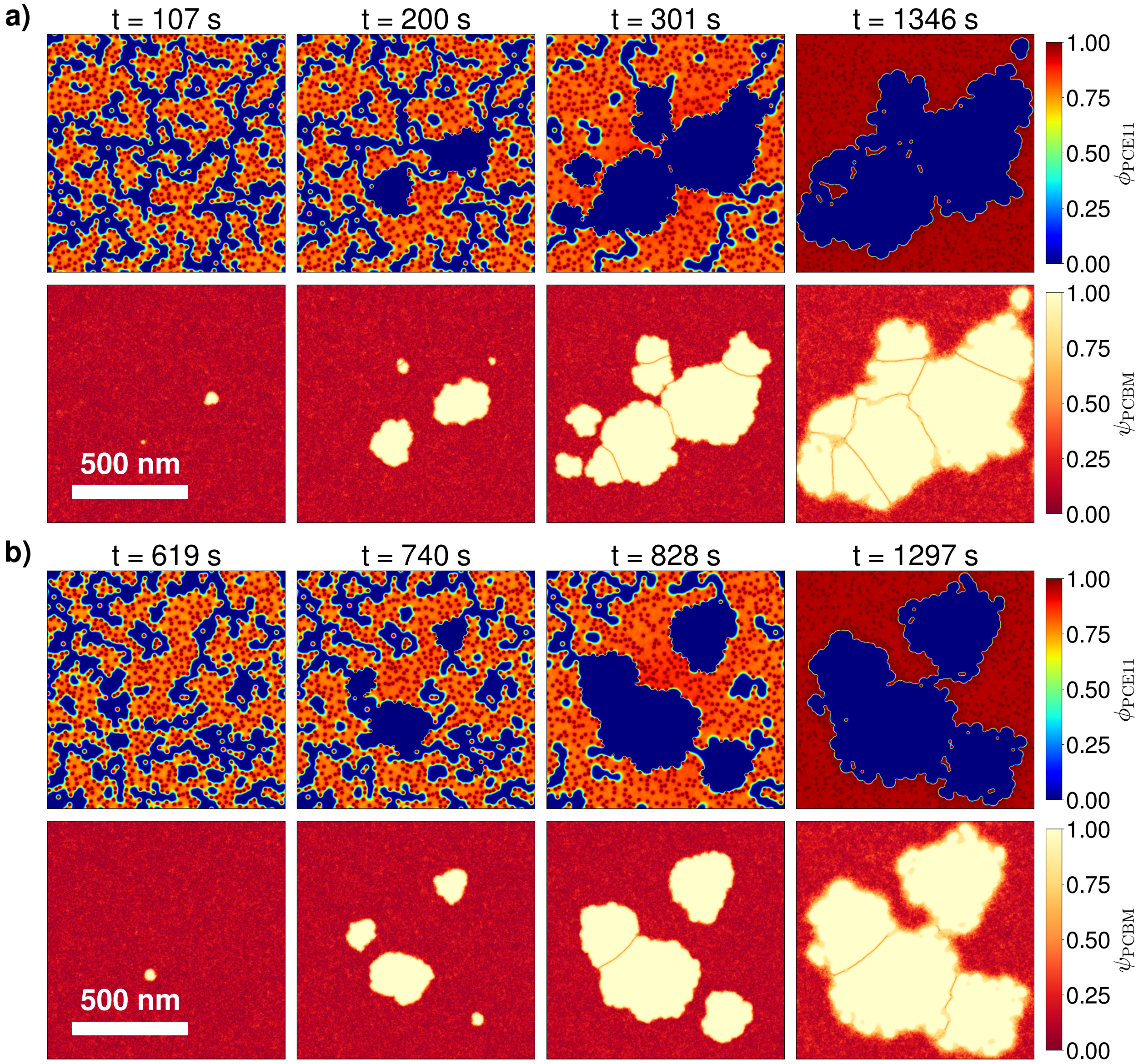

Figure 10: Simulations of PCE11:PCBM mixtures at T = 130 °C with randomly placed initial PCE11 crystallites and an increased PCBM crystal mobility coefficient M_{PCBM} = 5×10^{-2} s^{-1} (as compared to the case presented in the main body of the article). The diffusivity reduction coefficient in crystalline domains k_d is also set here to 5×10^{-5}. In a), the PCBM crystallization kinetics are accordingly accelerated. Due to the quench of the amorphous phase separation pattern by the PCE11 crystallites, the demixing-assisted PCBM crystallization still results in crystalline domains with similar geometries, crystal sizes, and grain numbers, independently of the timescale shift induced by the variation of M_{PCBM}. In b), the surface tension of PCBM crystals is additionally slightly increased with $\varepsilon^2_{\text{PCBM}}$ = 5.29×10^{-10} J/m. As a consequence, the nucleation time is delayed whereas the crystal growth speed is increased (i.e. the system becomes more growth-dominated), which affects the overall process kinetics and leads to less, but larger PCBM crystal grains. Apart from that, the global morphology evolution remains nevertheless comparable to the previous simulation case. All unspecified model parameters are those reported in the tables of SI-C. For each simulation, the upper and lower rows respectively show the evolution of the PCE11 volume fraction (ϕ_{PCE11}) and the PCBM order parameter (ψ_{PCBM}) fields.

References

1. Takaki, T. Phase-Field Modeling and Simulations of Dendrite Growth. *ISIJ International* **54,** 437–444 (2014).
2. Gránásy, L. *et al.* Phase-Field Modeling of Crystal Nucleation in Undercooled Liquids – A Review. *Progress in Materials Science* **106,** 100569 (2019).
3. Huggins, M. L. Theory of Solutions of High Polymers1. *Journal of the American Chemical Society* **64,** 1712–1719 (1942).
4. Flory, P. J. *Principles of Polymer Chemistry* (Cornell University Press, 1953).
5. Rubinstein, M. & Colby, R. H. *Polymer Physics* (Oxford University Press, 2003).
6. Matkar, R. A. & Kyu, T. Phase Diagrams of Binary Crystalline-Crystalline Polymer Blends. *The Journal of Physical Chemistry B* **110,** 16059–16065 (2006).
7. Matkar, R. A. & Kyu, T. Role of Crystal-Amorphous Interaction in Phase Equilibria of Crystal-Amorphous Polymer Blends. *The Journal of Physical Chemistry B* **110,** 12728–12732 (2006).
8. Saylor, D. M., Kim, C.-S., Patwardhan, D. V. & Warren, J. A. Diffuse-Interface Theory for Structure Formation and Release Behavior in Controlled Drug Release Systems. *Acta Biomaterialia* **3,** 851–864 (2007).
9. Kim, C.-S., Saylor, D. M., McDermott, M. K., Patwardhan, D. V. & Warren, J. A. Modeling Solvent Evaporation during the Manufacture of Controlled Drug-Release Coatings and the Impact on Release Kinetics. *Journal of Biomedical Materials Research Part B: Applied Biomaterials* **90B,** 688–699 (2009).
10. Ronsin, O. J. J. & Harting, J. Phase-Field Simulations of the Morphology Formation in Evaporating Crystalline Multicomponent Films. *Advanced Theory and Simulations,* 2200286 (2022).
11. Warren, J. A. & Boettinger, W. J. Prediction of Dendritic Growth and Microsegregation Patterns in a Binary Alloy Using the Phase-Field Method. *Acta Metallurgica et Materialia* **43,** 689–703 (1995).
12. Ronsin, O. J. J. & Harting, J. Formation of Crystalline Bulk Heterojunctions in Organic Solar Cells: Insights from Phase-Field Simulations. *ACS Applied Materials & Interfaces* **14,** 49785–49800 (2022).
13. Siber, M., Ronsin, O. J. J. & Harting, J. Crystalline Morphology Formation in Phase-Field Simulations of Binary Mixtures. *Journal of Materials Chemistry C* **11,** 15979–15999 (2023).
14. Shen, C., Simmons, J. & Wang, Y. Effect of Elastic Interaction on Nucleation: II. Implementation of Strain Energy of Nucleus Formation in the Phase Field Method. *Acta Materialia* **55,** 1457–1466 (2007).
15. Kramer, E. J., Green, P. & Palmstrøm, C. J. Interdiffusion and Marker Movements in Concentrated Polymer-Polymer Diffusion Couples. *Polymer* **25,** 473–480 (1984).
16. Levitsky, A. *et al.* Toward Fast Screening of Organic Solar Cell Blends. *Advanced Science* **7,** 2000960 (2020).
17. Levitsky, A., Schneider, S. A., Rabkin, E., Toney, M. F. & Frey, G. L. Bridging the Thermodynamics and Kinetics of Temperature-Induced Morphology Evolution in Polymer/Fullerene Organic Solar Cell Bulk Heterojunction. *Materials Horizons* **8,** 1272–1285 (2021).
18. Perea, J. D. *et al.* Introducing a New Potential Figure of Merit for Evaluating Microstructure Stability in Photovoltaic Polymer-Fullerene Blends. *The Journal of Physical Chemistry C* **121,** 18153–18161 (2017).
19. Li, N. *et al.* Abnormal Strong Burn-in Degradation of Highly Efficient Polymer Solar Cells Caused by Spinodal Donor-Acceptor Demixing. *Nature Communications* **8,** 14541 (2017).
20. Hajduk, B., Bednarski, H., Jarząbek, B., Janeczek, H. & Nitschke, P. P3HT:PCBM Blend Films Phase Diagram on the Base of Variable-Temperature Spectroscopic Ellipsometry. *Beilstein Journal of Nanotechnology* **9,** 1108–1115 (2018).
21. Zhang, C. *et al.* Comprehensive Investigation and Analysis of Bulk-Heterojunction Microstructure of High-Performance PCE11:PCBM Solar Cells. *ACS Applied Materials & Interfaces* **11,** 18555–18563 (2019).
22. Kim, J. Y. & Frisbie, C. D. Correlation of Phase Behavior and Charge Transport in Conjugated Polymer/Fullerene Blends. *The Journal of Physical Chemistry C* **112,** 17726–17736 (2008).

23. Zhao, J. *et al.* Phase Diagram of P3HT/PCBM Blends and Its Implication for the Stability of Morphology. *The Journal of Physical Chemistry B* **113,** 1587–1591 (2009).

24. Zhao, J. *et al.* Phase Behavior of PCBM Blends with Different Conjugated Polymers. *Physical Chemistry Chemical Physics* **13,** 12285–12292 (2011).

25. Machui, F., Rathgeber, S., Li, N., Ameri, T. & J. Brabec, C. Influence of a Ternary Donor Material on the Morphology of a P3HT:PCBM Blend for Organic Photovoltaic Devices. *Journal of Materials Chemistry* **22,** 15570–15577 (2012).

26. Ronsin, O. J. J. & Harting, J. Role of the Interplay between Spinodal Decomposition and Crystal Growth in the Morphological Evolution of Crystalline Bulk Heterojunctions. *Energy Technology* **8,** 1901468 (2020).

27. König, B., Ronsin, O. J. J. & Harting, J. Two-Dimensional Cahn–Hilliard Simulations for Coarsening Kinetics of Spinodal Decomposition in Binary Mixtures. *Physical Chemistry Chemical Physics* **23,** 24823–24833 (2021).
